# Gene expression in blood reflects smoking exposure among cancer-free women in the Norwegian Women and Cancer (NOWAC) postgenome cohort

**DOI:** 10.1038/s41598-020-80158-8

**Published:** 2021-01-12

**Authors:** Nikita Baiju, Torkjel M. Sandanger, Pål Sætrom, Therese H. Nøst

**Affiliations:** 1 Department of Community Medicine, Faculty of Health Sciences, UiT –the Arctic University of Norway, 9037 Tromsø, Norway; 2 Department of Computer Science, Norwegian University of Science and Technology, 7491 Trondheim, Norway; 3 Department of Clinical and Molecular Medicine, Norwegian University of Science and Technology, 7491 Trondheim, Norway; 4 Bioinformatics Core Facility, Norwegian University of Science and Technology, 7491 Trondheim, Norway; 5 Department of Public Health and Nursing, K.G. Jebsen Center for Genetic Epidemiology, Norwegian University of Science and Technology, 7491 Trondheim, Norway

**Keywords:** Gene expression, Risk factors

## Abstract

Active smoking has been linked to modulated gene expression in blood. However, there is a need for a more thorough understanding of how quantitative measures of smoking exposure relate to differentially expressed genes (DEGs) in whole-blood among ever smokers. This study analysed microarray-based gene expression profiles from whole-blood samples according to smoking status and quantitative measures of smoking exposure among cancer-free women (n = 1708) in the Norwegian Women and Cancer postgenome cohort. When compared with never smokers and former smokers, current smokers had 911 and 1082 DEGs, respectively and their biological functions could indicate systemic impacts of smoking. *LRRN3* was associated with smoking status with the lowest FDR-adjusted p-value. When never smokers and all former smokers were compared, no DEGs were observed, but *LRRN3* was differentially expressed when never smokers were compared with former smokers who quit smoking ≤ 10 years ago. Further, *LRRN3* was positively associated with smoking intensity, pack-years, and comprehensive smoking index score among current smokers; and negatively associated with time since cessation among former smokers. Consequently, *LRRN3* expression in whole-blood is a molecular signal of smoking exposure that could supplant self-reported smoking data in further research targeting blood-based markers related to the health effects of smoking.

## Introduction

Tobacco smoking is one of the major threats to public health, and it is currently responsible for more than 8 million deaths worldwide each year^1^. Exposure to tobacco smoke is a risk factor for many chronic diseases, such as cardiac and pulmonary diseases and several cancers. Further, smoking can suppress the immune system and modifies a range of immunological functions^2^. Subclinical outcomes, such as increased oxidative stress, reduced antioxidant defences, increased inflammation, impaired immune status, and altered lipid profiles, have been observed in smokers when compared to their counterparts who never smoked^3^. Notably, more respiratory symptoms caused by exposure to tobacco smoke have been observed in women than men^4,5^. Thus, tobacco smoking has several detrimental health effects, which might appear not long after smoking initiation or up to several decades after exposure^3,6^.

The toxic components of tobacco smoke are first absorbed in the lungs and then enter the blood stream before being distributed throughout the body, making blood an appropriate biological material to study the systemic influences of exposure to tobacco smoke^7^. In addition, the collection of whole-blood (or simply, ‘blood samples’) is easy and minimally invasive, and these samples can reveal features that are relevant for studies of human health effects^8^. Current exposure to tobacco smoke has been linked with modulated expression of many genes in blood, for example *LRRN3, CLDND1, GPR15, ATF4, SOD2,* and *CDKN1C*^9–16^. Altered gene expression in blood has also been linked to diseases for which smoking is a risk factor^17^. However, there is a need for a more thorough understanding of the variability in gene expression profiles in whole-blood in relation to quantitative measures of smoking exposure among ever smokers. Therefore, this cross-sectional analysis used data from 1708 cancer-free women participating in the prospective, population-based Norwegian Women and Cancer (NOWAC) postgenome cohort. Microarray-based gene expression profiles from bio-banked whole-blood samples were assessed according to smoking status and quantitative measures of smoking exposure (hereafter referred to as ‘smoking metrics’), such as smoking intensity, smoking duration, time since smoking cessation (TSC), pack-years, and comprehensive smoking index (CSI) scores^18^. Enriched pathways and gene ontology (GO) categories of significant genes associated with smoking were also assessed.

## Results

### General characteristics of the study population

The current study was based on microarray data from cancer-free women participating in the NOWAC postgenome cohort. The full cohort consists of approximately 50,000 women (mean age: 49.78 years; mean body mass index (BMI): 23.38 kg/m^2^), all of whom have given a blood sample. In total, 1708 of these women have been included as cancer-free controls in various studies and have gene expression profiles available for study, and only these women were included in the present analyses. All included women had completed up to three comprehensive questionnaires before blood collection (main questionnaires), and an additional questionnaire on lifestyle factors was completed at the time of blood collection. Thus, information was available for up to four time points in total. Smoking status and smoking metrics (smoking intensity, smoking duration, TSC, pack-years, and CSI scores) were based on information from all four questionnaires. Current smokers (CS) were defined as those who were currently smoking at the time of blood collection, former smokers (FS) were defined as those who reported smoking cessation prior to the time of blood collection, and never smokers (NS) were defined as those who reported they had never smoked either prior to or at the time of blood collection. CS and FS combined represented ever smokers. We defined passive smokers (PS) as those who were passively exposed to smoking at their homes as adults. Gene expression values were available for 7713 unique genes for all the women in this study.

We investigated associations between smoking status and potential covariates, such as age and BMI at blood collection, and white blood cell (WBC) proportions, using Chi-square or Kruskal–Wallis tests. We then performed a ‘global test’ to indicate any association between these variables and the overall gene expression data. We considered variables that were significant in both of these tests as potential confounders and adjusted for these in further models (Supplementary Table S1).

**Figure 1:**
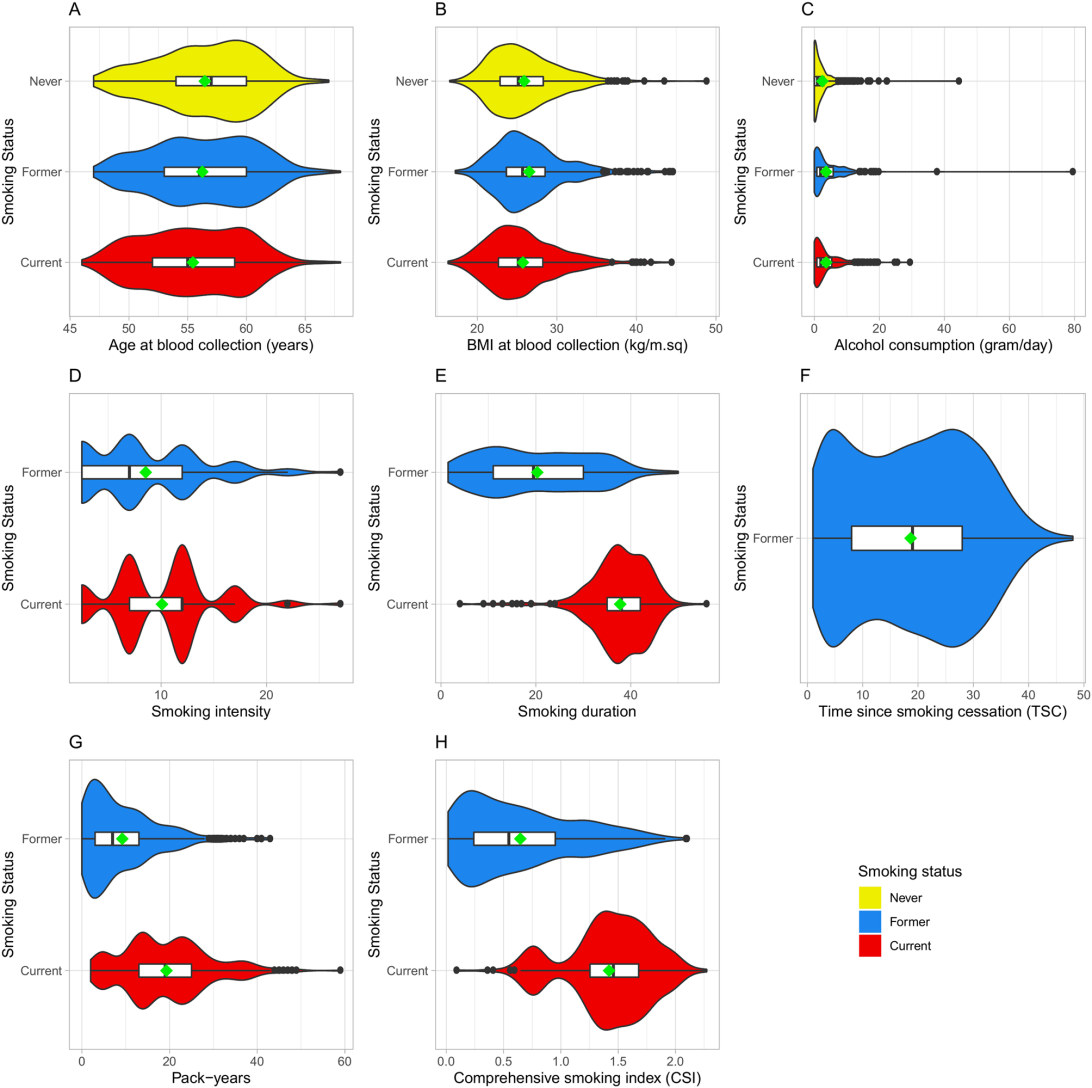
Descriptive statistics of study participants by smoking status for (**A**) age at blood collection, (**B**) body mass index (BMI) at blood collection, (**C**) alcohol consumption at baseline, (**D**) smoking intensity, (**E**) smoking duration, (**F**) time since smoking cessation (TSC), (**G**) pack-years, and (**H**) comprehensive smoking index (CSI) scores. Yellow, blue, and red coloured violin plots represent kernel density estimates for never, former, and current smokers, respectively. White boxes extend from the 25th to the 75th percentile, vertical bars inside the box represent the median, whiskers extend 1.5 times the length of the interquartile range right and left side of the 75th and 25th percentiles respectively, and outliers are represented as black dots. The green diamond shaped dot represents the respective mean.

There were 473, 613, and 622 CS, FS, and NS, respectively, among the 1708 women. The distributions of age and BMI at blood collection did not deviate markedly from normality, whereas the distribution of alcohol consumption was skewed (Fig. 1). Each of these distributions were similar across different categories of smoking status (Fig. 1A–C), but FS had the highest mean BMI and alcohol consumption, and NS had the highest mean age (Supplementary Table S1). Further, the smoking metrics—smoking intensity, smoking duration, pack-years, and CSI score had the highest means for CS as compared to FS (Fig. 1D–H). Finally, there were 192, 147, and 100 PS among CS, FS, and NS, respectively.

### Estimated white blood cell proportions

We estimated proportions of 22 types of WBCs using an in silico gene expression deconvolution method. CD8 T cells, naive CD4 T cells, resting NK cells, M0 macrophages, resting mast cells, and neutrophils were significantly associated with both smoking status and overall gene expression (Supplementary Table S2 and Supplementary Fig. S1). Further, we used linear regression to assess the associations between WBC proportions and smoking metrics. We observed that CD8 T cells were negatively associated with pack-years and CSI score; naive CD4 T cells were positively associated with smoking intensity, smoking duration, pack-years, and CSI score; resting NK cells were negatively associated with smoking intensity, smoking duration, pack-years, and CSI score but positively associated with TSC; resting mast cells were negatively associated with smoking duration; and neutrophils were negatively associated with TSC (Supplementary Table S3).

### Differentially expressed genes dependent on smoking status

We used two adjusted (minimally- and fully-adjusted) models to assess the relationships between smoking status and gene expression profiles, using the ‘limma’ package for gene-wise linear models. In minimally-adjusted models, we adjusted for technical variables such as laboratory batch (laboratory plates) and sample storage time, while in fully-adjusted models, in addition to the technical variables, we included the following variables that were associated with both the exposure and the outcome: selected WBC proportions, age, BMI, and use of hormone replacement therapy at the time of blood collection, as well as information on alcohol consumption and use of oral contraceptives, which was taken from the main questionnaires. The presence of differentially expressed genes (DEGs) was determined by three comparisons of smoking status groups: CS-vs-NS, CS-vs-FS, and FS-vs-NS. We considered Benjamini–Hochberg false discovery rates (FDR) with the significance threshold FDR ≤ 0.05.

**Figure 2:**
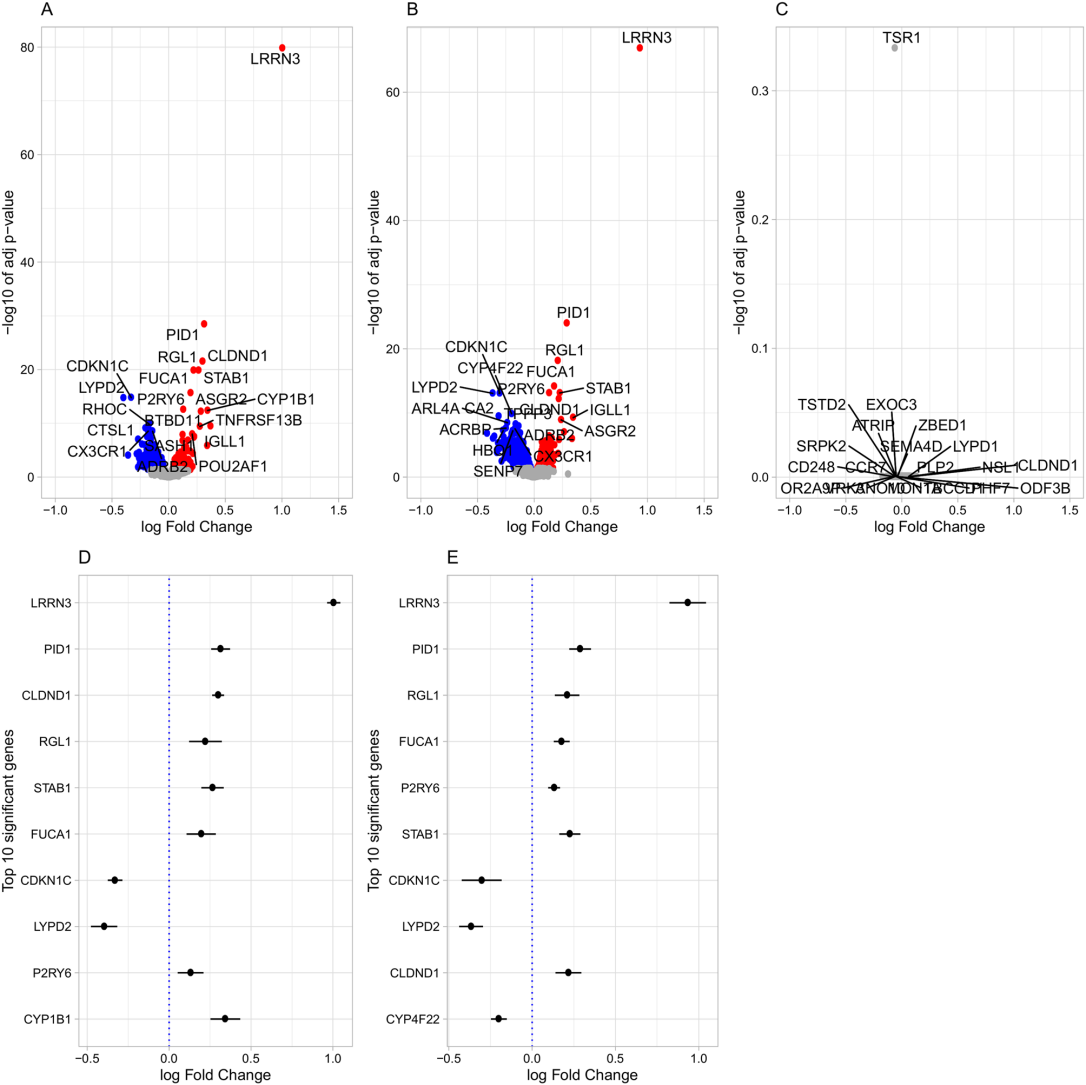
Volcano plots for the test statistics in fully-adjusted models from the tests of differentially expressed genes (DEGs) in comparisons of (**A**) current versus never smokers, (**B**) current versus former smokers, and (**C**) former versus never smokers; and forest plots for the 10 top-ranked DEGs in tests of DEGs in comparisons of (**D**) current versus never smokers and (**E**) current versus former smokers. In volcano plots (**A**–**C**), red dots display up-regulated genes, blue dots display down-regulated genes, while grey dots display genes with FDR > 0.05; the x-axis presents log_2_ fold-changes and the y-axis presents − log10 of FDR adjusted p-values; and gene names displayed are the 20 top-ranked DEGs in the respective tests. In forest plots (**D** and **E**), dots in the x-axis represent log_2_ fold-changes and the y-axis represents DEGS with the lowest FDR adjusted *p*-values ranked from the top; the horizontal line for each gene represents their confidence interval; and the vertical blue dotted line represents no difference.

In minimally-adjusted models, there were 1009 DEGs in the CS-vs-NS comparison; 427 up-regulated and 582 down-regulated genes. Correspondingly, in the CS-vs-FS comparison, there were 1371 DEGs (559 up-regulated, 812 down-regulated). In fully-adjusted models, there were 911 DEGs in the CS-vs-NS comparison (355 up-regulated, 556 down-regulated; Fig. 2A,D), and 1082 DEGs in the CS-vs-FS comparison (435 up-regulated, 647 down-regulated; Fig. 2B,E). The two adjusted models had 670 overlapping DEGs in the CS-vs-NS comparison (Supplementary Table S4) and 839 in the CS-vs-FS comparison (Supplementary Table S5). Similarly, the CS-vs-NS and CS-vs-FS comparisons had 776 and 652 overlapping DEGs in the minimally- and fully-adjusted models, respectively. In the fully-adjusted models, there were 230 up-regulated and 422 down-regulated genes that overlapped between the CS-vs-NS and CS-vs-FS comparison and displayed the same direction of effects. The top-ranked gene (i.e., the gene with the lowest FDR adjusted *p*-values) in all comparisons was *LRRN3* (Supplementary Fig. S2). Receiver operating characteristics (ROC) curve analyses showed that expression levels of *LRRN3*, as measured by the Illumina arrays, could strongly distinguish CS from NS and moderately distinguish FS (with ≤ 10 years TSC) from NS (Supplementary Fig. S3). Moreover, in a subset of our dataset, *LRRN3* expression showed similar discriminative power as DNA methylation at the *AHRR* CpG site (cg05575921), which is a known marker for smoking exposure^19^. There were no DEGs in the FS-vs-NS comparison in either model (Fig. 2C). However, *LRRN3* was the only DEG that remained significant when we included only FS with TSC ≤ 10 years and compared it with NS in the minimally-adjusted model (with log_2_ fold-change (logFC) = 0.34 and FDR = 3.63E−04). The *p*-values were uniformly distributed only in the FS-vs-NS comparison, but not in the other comparisons, as presented in quantile–quantile plots (Supplementary Fig. S4). Further, we used the ‘limma’ package to analyse the effects of passive smoking among NS, by contrasting all NS who were PS in adulthood with the other NS using the minimally-adjusted model. There were no DEGs when testing differences between PS (n = 100) and non-PS (n = 428).

### Analyses of smoking metrics within ever smokers

To identify genes associated with magnitude of smoking exposure, we used the ‘limma’ framework to identify genes for which the expression level correlated with the given smoking metrics among ever smokers. Specifically, we extended the minimally-adjusted model to include the given smoking metrics and analysed CS and FS separately.

**Figure 3:**
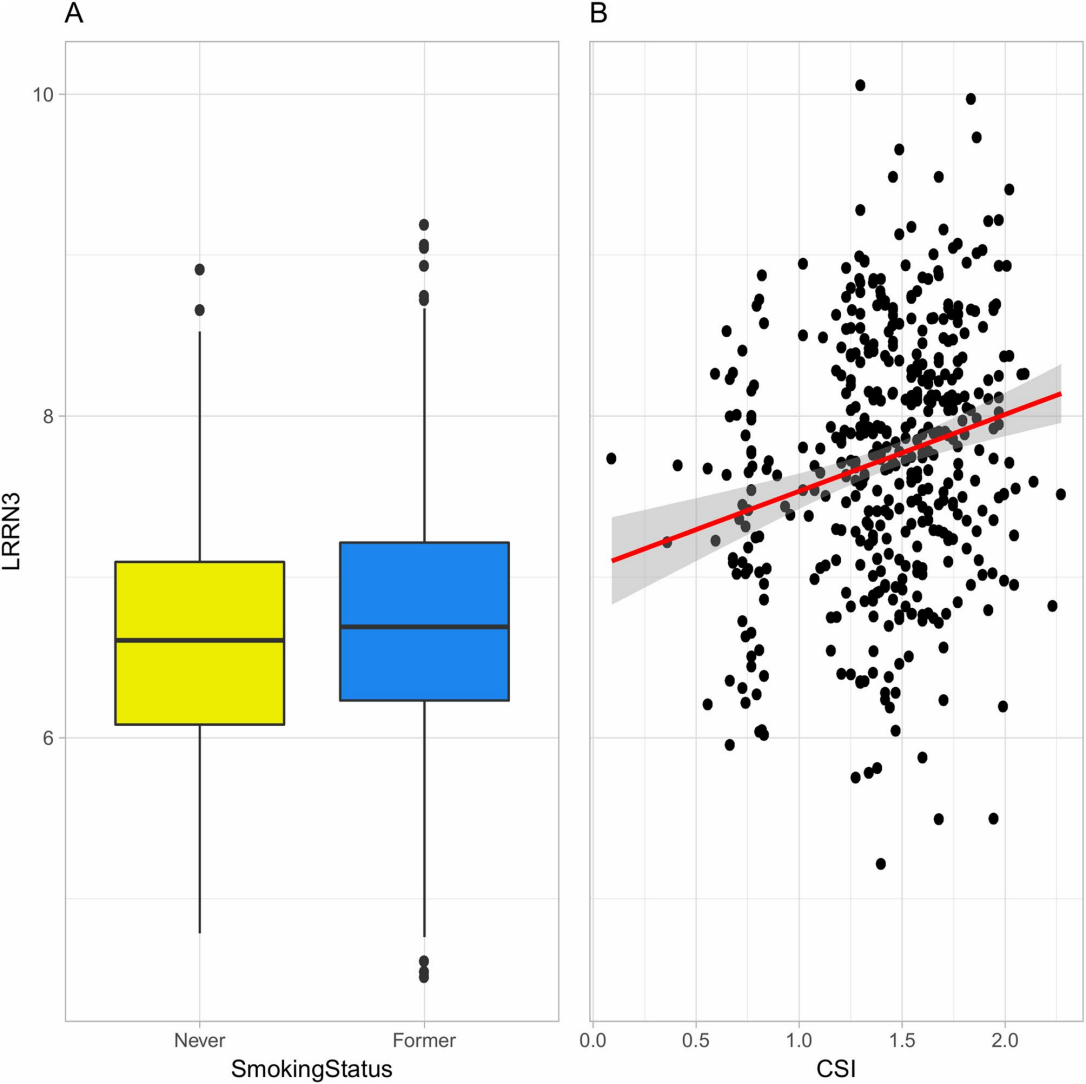
Distributions of expression values for the top-ranked gene (*LRRN3*) (**A**) among never (yellow) and former (blue) smokers and (**B**) among current smokers according to comprehensive smoking index (CSI) scores. In figure (**A**), boxes extend from the 25th to the 75th percentile, horizontal bars represent the median, whiskers extend 1.5 times the length of the interquartile range above and below the 75th and 25th percentiles, respectively, and outliers are represented as points. In figure (**B**), the red line represents the linear regression fit and the shaded grey area its standard error.

In analyses of CS, the top-ranked gene, *LRRN3* (logFC = 0.60, FDR = 4.70E−05), was positively associated with CSI score (Fig. 3). Further, there were five genes positively associated and two genes negatively associated with smoking intensity (Supplementary Table S6), where *LRRN3* was the top-ranked gene, with a positive association (Supplementary Fig. S5). Likewise, there were three genes positively associated and two genes negatively associated with pack-years (Supplementary Table S7), where *LRRN3* was the top-ranked gene, with a positive association (Supplementary Fig. S6). There were no genes significantly associated with smoking duration among CS.

**Figure 4:**
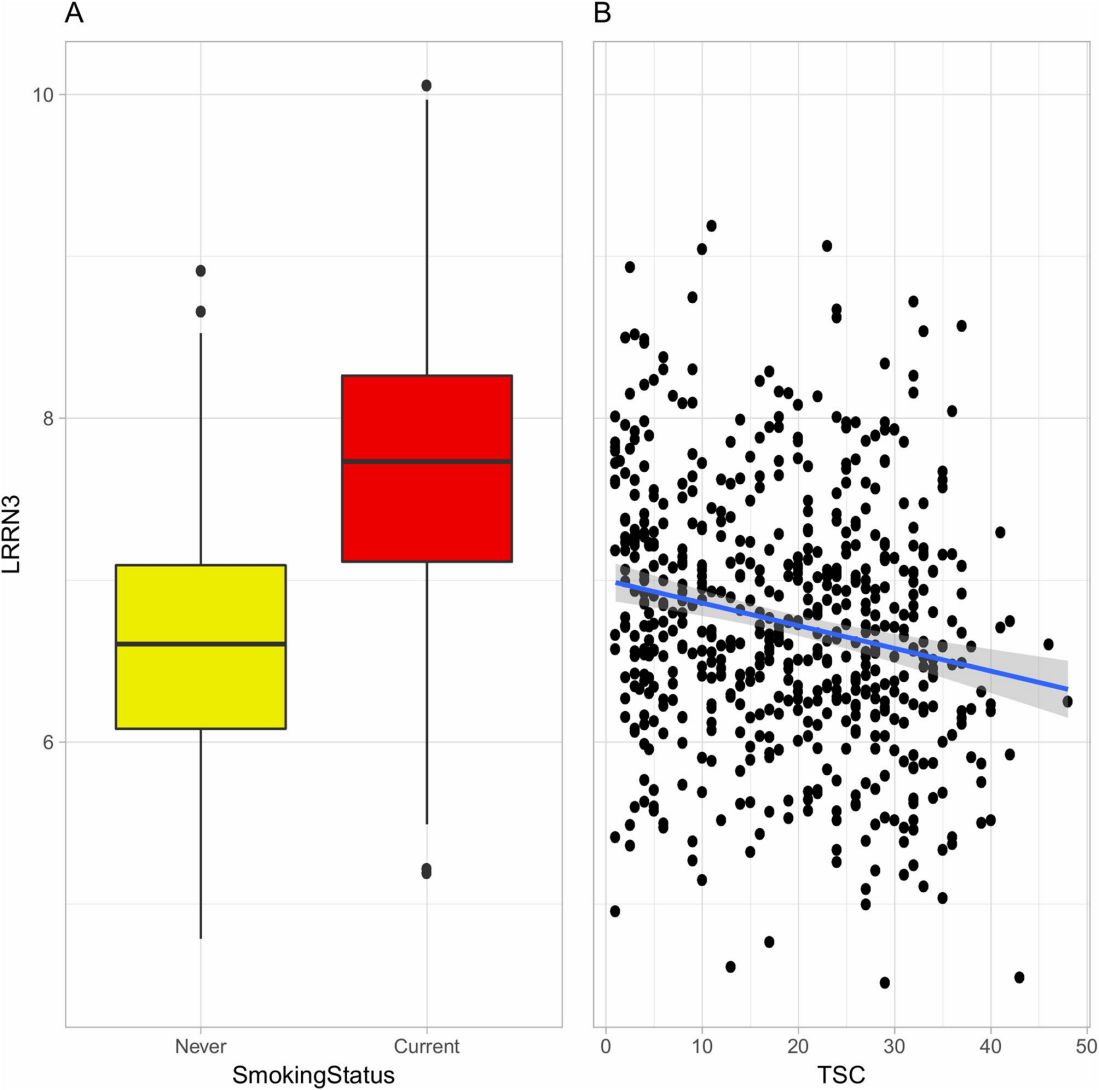
Distributions of expression values for the top-ranked gene (*LRRN3*) (**A**) among never (yellow) and current (red) smokers and (**B**) among former smokers according to time since smoking cessation (TSC). In figure (**A**), boxes extend from the 25th to the 75th percentile, horizontal bars represent the median, whiskers extend 1.5 times the length of the interquartile range above and below the 75th and 25th percentiles, respectively, and outliers are represented as points. In figure (**B**), the blue line represents the linear regression fit and the shaded grey area its standard error.

In analyses of FS, the top-ranked gene, *LRRN3* (logFC =  − 0.014, FDR = 2.63E−03), was negatively associated with TSC (Fig. 4). Correspondingly, *NMRAL1* (logFC =  − 0.008, FDR = 2.72E−02) was negatively associated with pack-years (Supplementary Fig. S7). No genes were significantly associated with smoking intensity, smoking duration, or CSI scores among FS.

### Functional enrichment analyses

**Figure 5:**
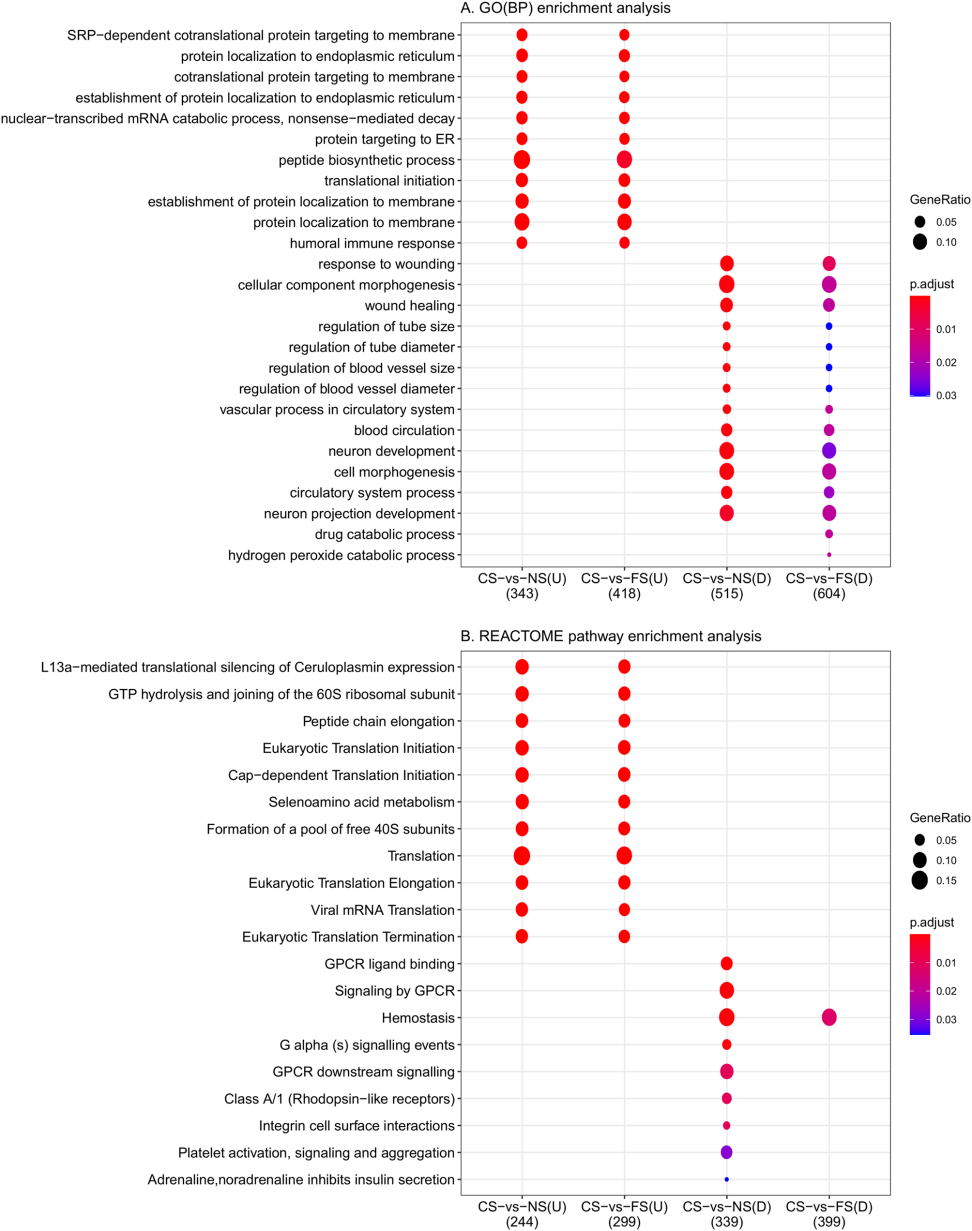
Summary of functional enrichment analyses for up- and down-regulated genes for the (**A**) GO(BP) and (**B**) REACTOME pathway databases. The colour of the dots indicates the adjusted p-value, where red dots represent the most enriched categories; the ‘GeneRatio’ indicates the proportion of genes overlapping between lists of differentially expressed genes (DEGs) and the genes in gene ontology categories. GO: gene ontology; BP: biological processes; CS-vs-NS: comparison of current smokers versus never smokers; CS-vs-FS: comparison of current smokers versus former smokers; U: Up-regulated genes; D: Down-regulated genes.

To investigate the potential common functions of the identified DEGs affected by smoking, we performed functional enrichment analyses to identify GO biological processes (BP), GO molecular functions (MF), GO cellular components (CC), Kyoto encyclopaedia of genes and genomes (KEGG) pathways, and REACTOME pathways enriched for DEGs in the CS-vs-NS and CS-vs-FS comparisons (Supplementary Tables S8–12, Fig. 5, and Supplementary Fig. S8). Analyses were performed for DEGs in fully-adjusted models and separately for up-regulated and down-regulated genes. The numbers of enriched terms in the respective categories are presented in Table 1.

**Table 1:** Number of enriched terms in different categories of enrichment analyses in comparisons of current versus never smokers (CS-vs-NS) and current versus former smokers (CS-vs-FS).

Database	CS-vs-NS		CS-vs-FS	
	Up-regulated genes (n = 355)	Down-regulated genes (n = 556)	Up-regulated genes (n = 435)	Down-regulated genes (n = 647)
GO(BP)	33	51	22	41
GO(MF)	4	0	6	5
GO(CC)	23	6	14	4
KEGG	1	7	1	0
REACTOME	31	9	34	1

For both up-regulated and down-regulated genes, enriched categories overlapped considerably for genes that were significant according to the FDR in the CS-vs-NS and CS-vs-FS comparisons. However, there were more enriched categories among genes in the CS-vs-NS comparison, except for GO(MF), where there were significant categories only in the CS-vs-FS comparison. Considering the terms themselves, up-regulated genes were mostly enriched for terms related to translation, such as ribosome (KEGG and GO(CC)), protein localisation to endoplasmic reticulum (GO(BP)), and translation (REACTOME). Terms were also related to immune responses, such as humoral immune response, inflammatory response, and B cell activation (GO(BP)). In contrast, down-regulated genes were enriched for many terms related to circulatory functions, including response to wounding and regulation of blood vessel size (GO(BP)), and extracellular signalling, such as G protein-coupled receptor ligand binding (REACTOME), and plasma membrane region (GO(CC)).

## Discussion

This study presents DEGs across categories of smoking status, as well as genes associated with different smoking metrics within ever smokers in the whole-blood of cancer-free women from the NOWAC postgenome cohort. These assessments, which used quantitative and repetitive smoking metrics, bring novel knowledge about the systemic responses to smoking exposure within ever smokers.

The study participants had similar proportions of CS, FS, and NS. They had comparable mean age and BMI at the time of blood collection as that of the full cohort, and to that of participants in other studies targeting the relation between smoking exposure and gene expression^9–11^. Among the 7713 genes assessed, 911 and 1082 genes were differentially expressed in CS-vs-NS and CS-vs-FS comparisons, respectively. When looking at the DEGs in the CS-vs-NS comparison and the significant genes indicated in corresponding tests in a large meta-analysis containing 10,233 participants (51% women), we found that among the 285 DEGs in our study that overlapped with the 1270 DEGs in that study, 282 genes had the same direction of effects^11^. Moreover, the mean expression levels for the 285 DEGs between CS-vs-NS that overlapped with DEGs identified in corresponding tests in the meta-analysis were higher (7.56) than those DEGs that did not overlap (6.83; t = 5.23, *p*-value = 2.63E−07). Still, the average absolute logFC for the overlapped (0.12) and non-overlapped DEGs (0.09; W = 128,066, *p*-value = 1.29E−14) were similar. This implies that the relation to smoking was consistent for hundreds of genes between these studies and demonstrates the comprehensive effects of smoking on gene expression in blood.

Around 40% of the genes were over-expressed in CS as compared to both NS and FS (i.e., 60% were under-expressed). Although higher proportions of up-regulated genes have been observed more frequently in other studies^7,9–11^, higher proportions of down-regulated genes have also been observed^14,16^. Interestingly, there could be sex differences in the directionality of observed DEGs, as one study comparing smokers and non-smokers observed that 29% of DEGs in men were down-regulated, compared to 62% in women^7^. However, only about 4% of the DEGs in our study were in X-chromosomes in both the CS-vs-NS and CS-vs-FS comparisons. Notably, differences in gene expression between adult men and women do not need to originate in genes on the X-chromosomes, but a meta-analysis of sex expression differences in blood found that 25% of DEGs do map to the sex chromosomes^20^. Thus, it is unlikely that the higher proportion of down-regulated genes in our study was due to the inclusion of women only.

Among NS, there were no genes associated with self-reported passive smoking in their homes as adults when compared to individuals with no passive smoking exposure. This could indicate that gene expression was more influenced by tobacco smoking of the women themselves. However, this could also be due to lack of statistical power or an imprecise exposure measure (lack of detailed information on timing, duration, and intensity of exposure).

Among CS, there were one, five, and three significant genes that were positively associated with CSI scores, smoking intensity, and pack-years, respectively. Among these, the top-ranked gene, *LRRN3,* was up-regulated in CS, which demonstrated that even within CS, *LRRN3* had a higher expression among those with a higher smoking exposure, as represented by increasing CSI scores, smoking intensity, and pack-years. Among FS, there was one significant gene that was negatively associated with TSC (*LRRN3*) and one that was negatively associated with pack-years (*NMRAL1*). This demonstrated that within FS, those who had quit smoking recently had a higher expression of *LRRN3* than those who had quit long ago, and FS with more pack-years had a lower expression of *NMRAL1* than those with fewer pack-years. Also, when restricting the FS-vs-NS comparison to recent quitters (with TSC ≤ 10 years), *LRRN3* remained significant in minimally-adjusted models. This indicates that there are differences in gene expression related to ongoing smoking exposure in women that persist for *LRRN3* in those who recently stopped smoking. Thus, *LRRN3* expression increases during smoking exposure and years after smoking cessation, but it eventually reverts back to levels similar to those of NS. However, according to the results of our linear model (Fig. 4), it appeared to take approximately 20–30 years for *LRRN3* expression in FS to reach the average expression among NS. The difference in results from the overall FS-vs-NS comparison and those restricted to FS with TSC up to 10 years emphasises that TSC needs to be taken into account when analysing smoking effects in FS.

*LRRN3* was the top-ranked DEG in most comparisons, and its expression differences were large compared to the other DEGs identified. *LRRN3* has been consistently indicated to be over-expressed in the whole-blood of CS or FS in previous studies^9–11,13,14,16,21,22^. This gene is highly expressed in the adrenal glands, the brain, and the lungs, but also in 11 other tissues^23^, and *LRRN3* codes for a membrane protein. The GO database has little information on *LRRN3*′s potential functions, except that electronic annotations indicate that it is involved in the positive regulation of synapse assembly^23,24^. Notably, *LRRN3* has six known SNPs^25^ but genetic variants in participants were not available in this study. Top-ranked DEGs other than *LRRN3* in the CS-vs-NS and CS-vs-FS comparisons were *PID1, RGL1*, and *STAB1*, and in the analyses of ever smokers was *NMRAL1*. These genes are expressed in various tissues that differed across genes. The main functions of the aforementioned genes are to increase the proliferation of pre-adipocytes (*PID1*)^26^; to be involved in probable guanine nucleotide exchange factor (*RGL1*)^27^; and to act as a scavenger receptor for acetylated low-density lipoprotein, bind to both gram-positive and gram-negative bacteria, and to play a role in the defence against bacterial infection (*STAB1*)^28^. However, the interpretation of the potential function of these genes in blood in relation to smoking is not clear.

We performed functional enrichment analyses for GO(BP), GO(MF), and GO(CC) categories; and for KEGG and REACTOME pathways. This gave insight into the underlying biology and provided knowledge of pathways for the identified DEGs^29^. The overlap in the enriched categories of the up-regulated and down-regulated genes in the CS-vs-NS and CS-vs-FS comparisons indicated that similar GO categories and pathways were enriched when current smoking exposure was compared to both FS and NS. Still, the enrichment was clearer when CS were compared to NS than to FS. The latter might be because the effect of smoking was not completely absent or was being slowly reduced in FS. In addition, the overall lack of overlap for enriched categories of the up-regulated and down-regulated genes likely demonstrated that these separate groups of genes are involved in different pathways.

The GO enrichment analysis indicated categories such as peptide metabolic and biosynthetic processes, protein formation and translation, humoral immune response, structural constituent of ribosome and molecule activity, ribosomal subunits, and adherens junction were up-regulated in CS. In contrast, processes such as response to wounding, circulatory system, regulation of blood vessels and tube size and diameter, neuron projection development, drug and hydrogen peroxide catabolic processes, heme binding, cell body, and hemoglobin complex were down-regulated. Categories indicated in the KEGG and REACTOME enrichment analyses were largely in line with those in GO analysis. In summary, these categories indicate that the DEGs we identified were enriched for functions related to the physiological effects of smoking on the human body, which are well documented in the literatures. This is particularly relevant for the physiological functions linked to the cardiovascular system, as DEGs measured in blood could be directly influenced by such altered functions. For example, carbon monoxide binds to haemoglobin, thereby reducing the blood’s oxygen-carrying capacity^30^. Accordingly, our results indicated that smoking could also down-regulate genes involved in the haemoglobin complex, thereby potentially exacerbating smoking’s negative effects on oxygen transport. Further, smoking causes several negative vascular effects, including decreased coronary blood flow and myocardial oxygen delivery, as well as adverse effects on lipids, blood pressure, and insulin resistance^31^. Thus, the down-regulated processes for blood vessel size and diameter, and vascular processes in the circulatory system. The general circulatory system processes indicated in whole-blood in this study could be related to these known physiological effects of smoking. We identified that oxidoreductase activity was down-regulated, which is in line with smokers experiencing measurable and immediate oxidative damage, resulting in oxidative stress^3^. We also observed down-regulated wound healing and haemostasis, which is in agreement with observations of a reduced capacity to heal wounds among smokers^3,30^. Lastly, categories related to immune responses were up-regulated in CS. Smoking can compromise the immune system and immune homeostasis as a whole^3^, and gene enrichment analyses of genes related to smoking in other studies have indicated effects on the regulation of immune system processes^9,10,13–16^. GO analyses in a large meta-analysis of genes related to smoking demonstrated enrichment mainly for activation of platelets and lymphocytes, immune response, and apoptosis^11^. The enriched terms for the DEGs in our study only were largely the same as for those for DEGs that overlapped between the meta-analysis and our study (results not presented). Further, the expression of *LRRN3* has been linked to the methylation of a CpG site on the *AHRR* gene^19^ and *AHRR* is linked to AHR and CYP proteins, which represent detoxifying mechanisms in the liver. This can be a plausible physiological influence of smoking exposures. Still, considering the great variety of molecules in tobacco smoking, it can potentially influence multiple pathways, which was observed in the GO categories indicated.

In general, gene expression profiles in whole-blood are affected by the underlying composition of WBCs in the respective samples. Thus, skewed WBC proportions could act as confounders when identifying gene expression differences related to exposures like smoking, which can disturb WBC populations^16^. Neutrophils constituted a large fraction of estimated WBCs but was considerably lower as estimated from gene expression than what is typical in blood^32,33^ as well as estimated from DNA methylation in a subset of the samples (n = 324)^19^. Still, we observed that WBC proportions and smoking metrics—especially resting NK cells but also CD8 T cells, resting mast cells, and neutrophils—were negatively associated with increasing smoking exposure. Further, naive CD4 T cells were positively associated with several smoking metrics. These results are in line with observations that smoking may have detrimental effects on the immune capacity of the body. Indeed, smoking has been shown to be a significant and reversible cause of elevated WBC counts in healthy adults^34^. These estimated cell proportions were included in our fully-adjusted models when assessing DEGs. Still, the top-ranked genes identified in fully-adjusted models were similar to those from the minimally-adjusted models, indicating that these genes were likely not substantially confounded by the distributions of WBC.

The main strength of this study was its use of smoking metrics based on detailed, repeated information on past and recent smoking history of the study participants when assessing DEGs in blood between smoking status groups. Among the women we included in our study, 51%, 24%, and 25% had information available at four, three, and two time points, respectively. Still, this study was based on self-reported smoking information from questionnaires, as in most other studies^9,10,13–16^. Many studies have measured concentrations of the metabolite of nicotine, cotinine, in blood, urine or saliva in addition to self-reported smoking status^9,14–16^. However, due to its relatively short half-life (16–19 h)^35^, it would not have provided valuable information for FS. Further, DNA methylation at specific CpG sites have also showed promising abilities as markers of smoking status and could reflect smoking exposures even decades after cessation^36,37^. In a subset of our data, *LRRN3* demonstrated similar ability to discriminate CS and FS (with ≤ 10 years TSC) from NS as compared to methylation at the CpG cite in the *AHRR* gene. Therefore, the abilities of *LRRN3* expression as a quantitative marker for discrimination of smoking status should be investigated in other population samples and with the comparison to other markers.

This study comprised a large number of women (n = 1708), whereas most studies targeting associations between smoking exposure and gene expression in blood have been conducted in rather small samples, ranging from 9 to 219 participants^9,10,13–15^. The two exceptions are one population-based cohort study in the Netherlands with 3319 participants (65% women)^16^ and a meta-analysis with 10,233 participants (51% women)^11^. As mentioned, our results are in line with those observed in these studies. The present study included only cancer-free women, although we cannot disregard influences of other common chronic diseases. Further, this study was based on whole-blood samples, which is a relevant tissue to investigate the effects of smoking, as it expresses a large proportion of the genes in the human genome^16^. Still, the current cross-sectional study results represent snapshots of gene expression in blood^38^. Lastly, although RNA-sequencing has become a routinely used technology, results from microarray technology, like those in this study, are still reliable and overall comparable to RNA-sequencing results^39^. However, RNA-sequencing technology would be relevant for studying the effects of smoking exposure on other genes not captured by the Illumina microarray technology, such as most non-coding RNAs.

In conclusion, our results demonstrated associations between smoking exposure and gene expression profiles in whole-blood of cancer-free women in the NOWAC postgenome cohort. The use of quantitative, reliable, and repeated measurements of past and recent smoking exposures was the novelty of this study, as it contributes new knowledge on systemic responses of smoking exposure. Close to a thousand DEGs in comparisons between CS and NS or FS, *LRRN3*, was the top-ranked gene. *LRRN3* was also associated with CSI score, smoking intensity, and pack-years among CS; and with TSC among FS. Consequently, *LRRN3* expression in blood is a molecular signal of smoking exposure that could supplant self-reported smoking data in gene expression studies of the association between smoking exposure and specific phenotypes. The biological functionality of the DEGs identified were linked to circulatory functions, translation, and immune responses, and could indicate systemic impacts of smoking. Genes that are differentially expressed depending on smoking exposure could be of interest in studies that focus on the effects of smoking exposure on health. This study has provided knowledge on the relationship of genes and pathways with detailed information on smoking exposure among cancer-free women.

## Methods

### Study population

The NOWAC study is a nation-wide, population-based prospective cohort study initiated in 1991. Currently, it includes approximately 172,000 Norwegian women aged 30–70 years. Women were randomly selected from the Norwegian National Population Register and sent an invitation letter along with a first questionnaire, which included a detailed set of questions related to smoking exposure, height, weight, reproductive history, hormone replacement therapy, alcohol consumption, family history of breast cancer, dietary patterns, use of medication, and others. Since then, each woman has answered between one and three follow-up questionnaires (main questionnaires). The NOWAC study database takes information from the Cancer Registry of Norway, as well as national death and emigration registries. Details about the NOWAC study are available in Lund et al.^40^.

The current study was based on data from the NOWAC postgenome cohort^41,42^, a sub-cohort of the NOWAC study. This consists of approximately 50,000 women who, between 2003 and 2006, had blood samples collected in PreAnalytiX (PAX) gene-tubes for gene expression analysis and, at the same time, answered a less extensive questionnaire about their lifestyle. The current study incorporated microarray-based expression profiles in bio-banked whole-blood samples from cancer-free women in the NOWAC postgenome cohort, who were originally enrolled as controls in several studies on breast, lung, ovarian, and endometrial cancers, and diabetes. We obtained relevant questionnaire and registry information from NOWAC databases and excluded those women that did not respond to any questions on smoking exposure, those who participated in more than one study, and those who were diagnosed with cancer before 2017. This resulted in a final analytical sample of 1708 women.

### Smoking status and smoking metrics

The main questionnaires included detailed questions regarding past and current smoking exposures, including ages at smoking initiation and cessation, average number of cigarettes smoked per day across age intervals, and details about passive smoking. Smoking status and smoking metrics (smoking intensity, smoking duration, TSC, pack-years, and CSI scores) were based on information from all main questionnaires and the questionnaire completed at the time of blood collection. Smoking intensity was defined as the average number of cigarettes smoked per day during years of active smoking, smoking duration was the duration of active smoking in years, and TSC was the time since smoking cessation in years. Pack-years quantify individual, long-term exposure to tobacco smoking^43^; this variable was calculated by the formula: *Number of pack-years* = *(smoking intensity/20)* × *smoking duration.* We considered 20 cigarettes in 1 pack, which is standard in the Norwegian context. CSI score is a cumulative measure of smoking exposure that incorporates smoking intensity (int), smoking duration (dur), and TSC (tsc). CSI scores were calculated using the formula^18^: *CSI* = (1 − 0*.*5^dur∗*/τ*^*)(*0*.*5^tsc∗*/τ*^*)* ln(int + 1), where τ is an estimated half-life parameter, and δ is an estimated lag time parameter describing TSC and total duration as follows:

$$ tsc^{*}\, = \,max\left( {tsc\, - \,\delta ,\,0} \right)\,{\text{and}}\,dur^{*}\, = \,max\left( {dur\, + \,tsc\, - \,\delta } \right)\,{-}\,tsc^{*}. $$

### Laboratory analyses and pre-processing of the gene expression data

Total RNA was extracted and purified from PAX gene-tube samples according to the PAX gene blood RNA kit protocol at the Genomics Core Facility, Norwegian University of Science and Technology (NTNU), Trondheim. A NanoDrop ND 8000 spectrophotometer (ThermoFisher Scientific, Wilmington, DE, USA) was used to assess RNA purity, and bio-analyser capillary electrophoresis (Agilent Technologies, Palo Alto, CA, USA) was used to assess RNA integrity. Complementary RNA (cRNA) was prepared using the Illumina TotalPrepT-96 RNA amplification kit, and hybridised to Illumina human WG-3 or HT-12 expression bead chip microarrays. The raw microarray images were processed in Illumina genome studio. The laboratory analysis date varied from January 2011 to January 2015.

For each study sample set separately, potential outliers were evaluated based on plots such as principal component analysis (PCA) plots and boxplots of probe signals displaying variation along with the laboratory quality measures^44^. We performed background correction, removed bad quality probes, and filtered probes detected in less than 20% of samples. Further, we performed log_2_ transformation and quantile normalisation before all data were combined and inspected for batch effects using PCA plots. We performed gene annotation using the Bioconductor packages *‘lumi’, ‘lumiHumanIDMapping’*, and *‘illuminaHumanv4.db’*^45–47^. If there were more than one probe annotated to each gene, the probe with the largest inter-quartile range was kept, which resulted in 7713 unique genes in the data analysed. Estimates for the proportions of 22 populations of WBCs in samples were obtained using the CIBERSORT procedure^48^.

### Statistical analyses

We considered covariates and WBC proportions as potential confounders if they were significantly associated with smoking status according to Chi-square or Kruskal–Wallis tests, and with overall gene expression data according to the ‘global test’ from the Bioconductor package *‘global test’*^49^. We used two adjusted (minimally- and fully-adjusted) models to assess the relationship between smoking status and gene expression profiles. We also performed linear regression analysis between WBC proportions and smoking metrics to assess their associations.

We performed all the main analyses using R version 3.2.1 and 3.6.2^50^. We used the Bioconductor package ‘*limma*’^51^ for the gene-wise linear models. The presence of DEGs was determined by three comparisons of smoking status groups: CS-vs-NS, CS-vs-FS, and FS-vs-NS, using a significance threshold of FDR ≤ 0.05^52^. We performed analyses of smoking metrics within CS and FS separately, and for adult PS within NS. Further, data on DNA methylation at the CpG site *AHRR* gene, cg05575921, was available in a subset of participants (n = 324)^19^. Therefore, we compared the ability of the top-ranked gene in our analyses and CpG site in the *AHRR* gene (cg05575921) using ROC curves. Differences in average expression and log_2_FC between groups of DEGs were tested using t-test and Wilcoxon rank sum test, respectively. To evaluate common biological functions of results of the gene-wises tests, we performed functional enrichment analyses of all significant up-regulated genes and all significant down-regulated genes. We used the bioconductor packages *‘clusterProfiler’*^53^ and ‘*ReactomePA*’^54^ to conduct functional enrichment analyses of GO(BP), GO(MF), and GO(CC) categories, and KEGG^55^ and REACTOME pathways for DEGs from different smoking status groups.

### Ethical statement

The Regional Ethical Committee of North Norway (REK) has approved the NOWAC study and the NOWAC postgenome cohort (Reference Numbers: 2010/2075/REK Nord and 2014/1605/REK Nord, respectively), and the collection and storage of human biological material, the individual case–control studies, and gene expression analyses that this project was constructed from. The women gave written informed consent for the blood collection and for gene expression analyses^42^. All methods were carried out in accordance with relevant guidelines and regulations in the manuscript for human.

## Supplementary Information

Supplementary Figures.

Supplementary Tables.

## Data Availability

Data cannot be shared publicly because of local and national ethical and security policy. Data access for researchers will be conditional on adherence to both the data access procedures of the Norwegian Women and Cancer Cohort and the UiT –The Arctic University of Norway (contact via Tonje Braaten <tonje.braaten@uit.no> and Arne Bastian Wiik <arne.b.wiik@uit.no>) in addition to an approval from the local ethical committee.
