# Supplementary material for: Gene expression in blood reflects smoking exposure among cancer-free women in the Norwegian Women and Cancer (NOWAC) postgenome cohort
Source: Sci Rep. 2021 Jan 12;11:680. doi: 10.1038/s41598-020-80158-8 (PMC7803754; doi:10.1038/s41598-020-80158-8)
Supplement: Supplementary file 1 — Supplementary Figures. [file 41598_2020_80158_MOESM1_ESM.pdf]

Gene expression in blood reflects smoking exposure among cancer-free women in the Norwegian Women and Cancer (NOWAC) postgenome cohort

Nikita Baiju[1,*], Torkjel M. Sandanger[1], Pål Sætrom[2, 3, 4, 5], and Therese H. Nøst[1, 5]

Supporting information

Supplementary Figures

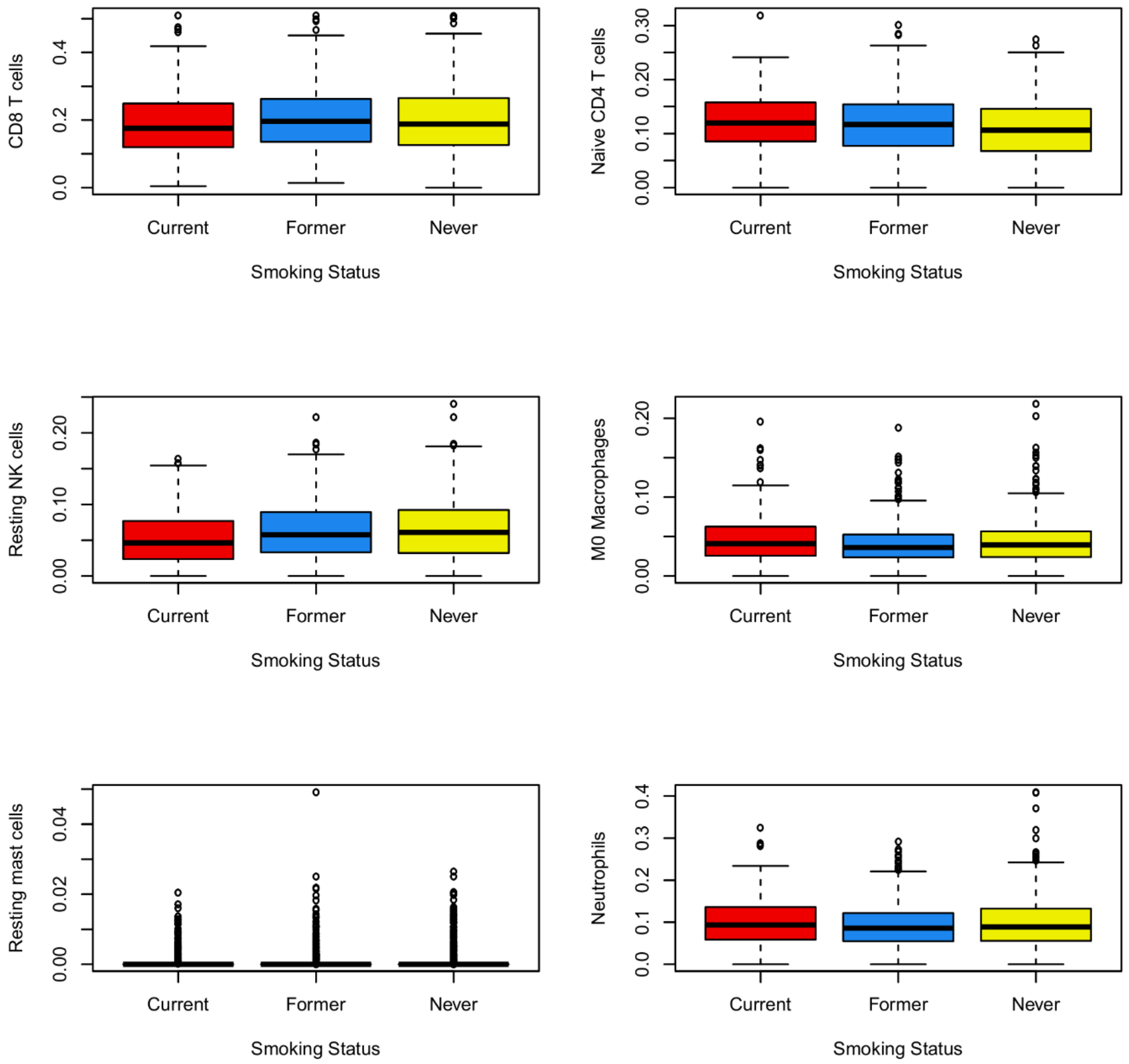

Supplementary Figure S1. Boxplots for white blood cell (WBC) proportions that were different among current smokers (red), former smokers (blue), and never smokers (yellow). The X-axis displays the smoking status and the Y-axis displays the proportions of WBCs. Boxes extend from the 25th to the 75th percentile, horizontal bars represent the median, whiskers extend 1.5 times the length of the interquartile range above and below the 75th and 25th percentiles, respectively, and outliers are represented as small circles. (TIFF)

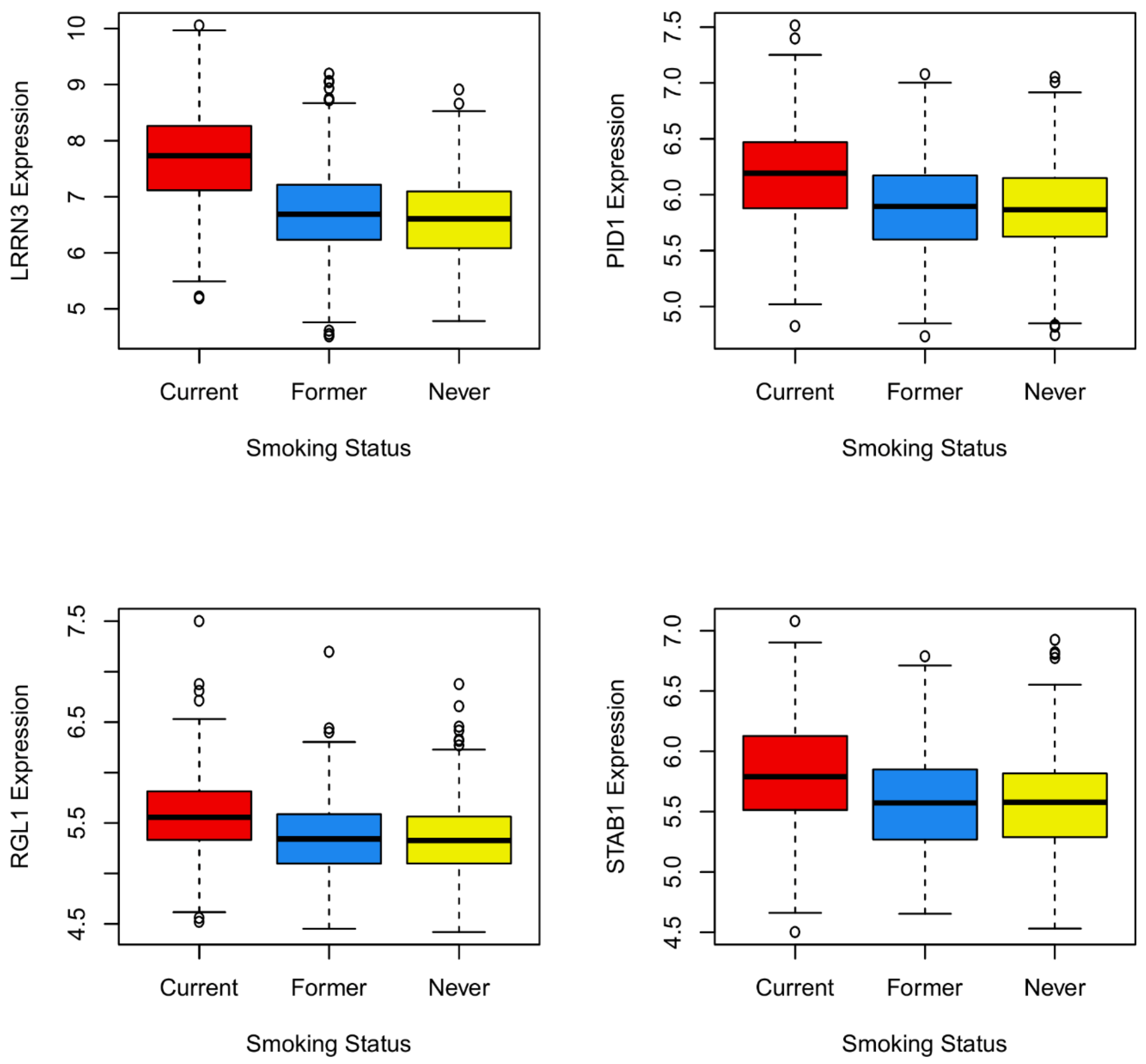

Supplementary Figure S2. Boxplots for expression values of the four top-ranked genes in comparisons of current vs never smokers, and current vs former smokers that were different among current smokers (red), former smokers (blue), and never smokers (yellow). The X-axis displays the smoking status and the Y-axis displays the gene expression values. Boxes extend from the 25th to the 75th percentile, horizontal bars represent the median, whiskers extend 1.5 times the length of the interquartile range above and below the 75th and 25th percentiles, respectively, and outliers are represented as small circles. (TIFF)

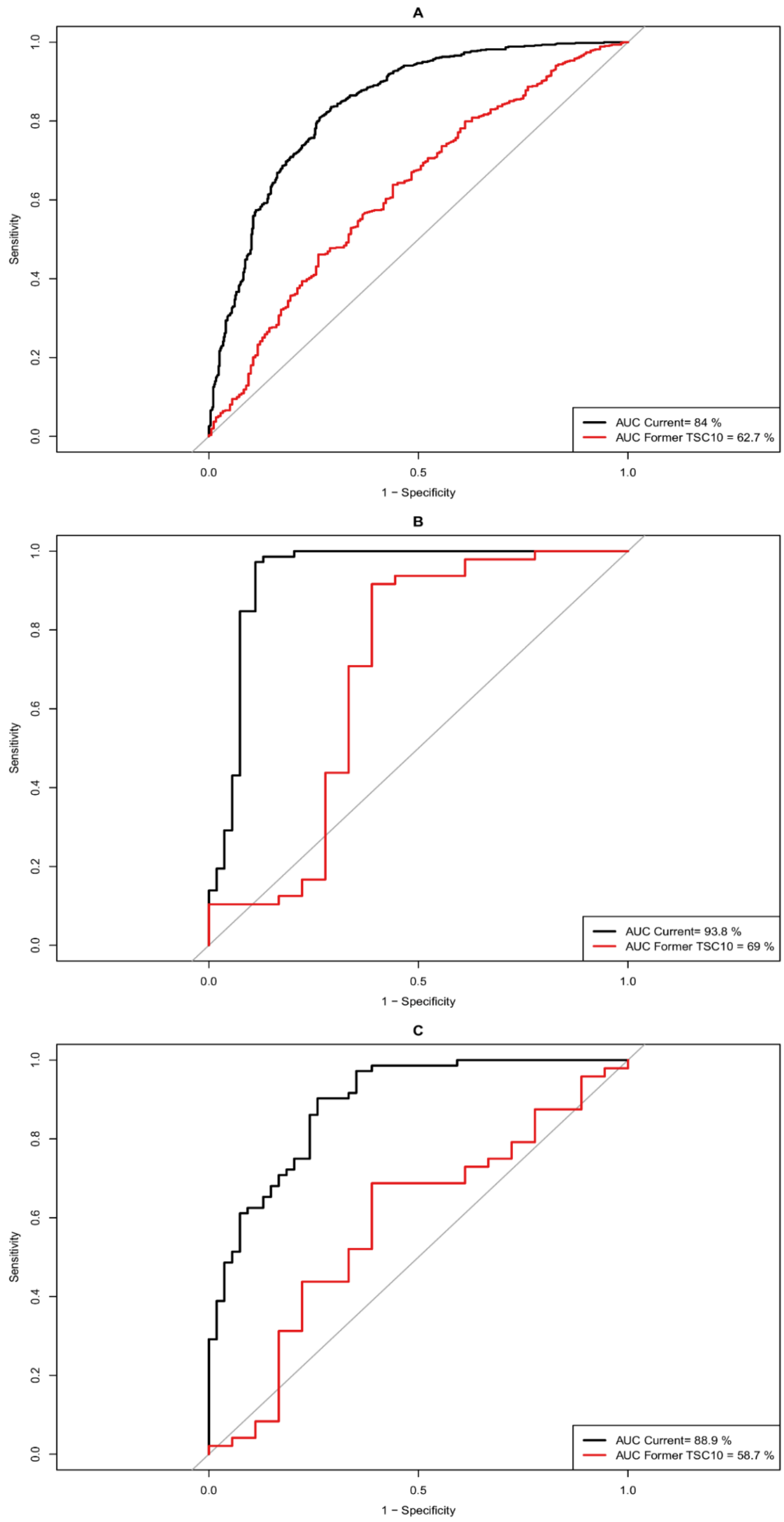
A
AUC Current= 84 %
AUC Former TSC10 = 62.7 %
B
AUC Current= 93.8 %
AUC Former TSC10 = 69 %
C
AUC Current= 88.9 %
AUC Former TSC10 = 58.7 %
Sensitivity
1 − Specificity

Supplementary Figure S3. Receiver operating characteristics (ROC) curve for (A) presents the ability of *LRRN3* to discriminate between current from never smokers (black line, n=1095) and former (with TSC≤10 years) smokers from never smokers (red line, n=810), (B) presents the ability of cg05575921, a CpG on the *AHRR* gene, for the same discrimination in a subset of samples n=126 and 74, respectively), and (C) presents the ability of *LRRN3*, for the subset of samples that had DNA methylation data available (n=126 and 74, respectively), for the same discrimination. The X-axis presents the specificity of the model and the Y-axis presents the sensitivity.

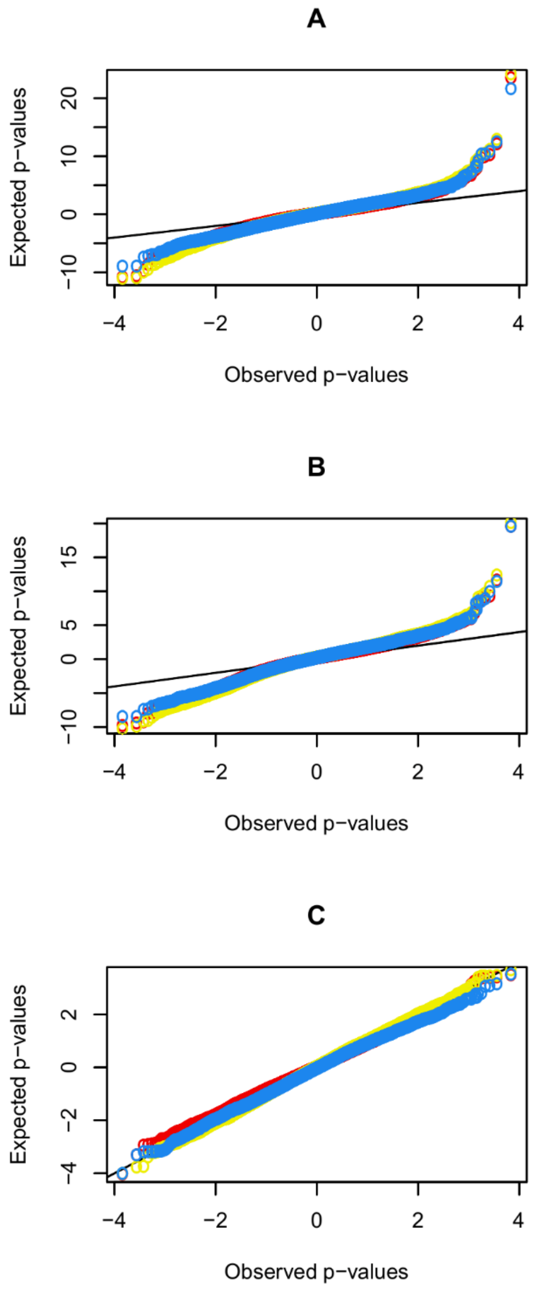

Supplementary Figure S4. Quantile-quantile plots for comparisons of (A) current vs never smokers, (B) current vs former smokers, and (C) former vs never smokers in unadjusted models (red), minimally-adjusted models (yellow), and fully-adjusted models (blue). The X-axis shows the ‘observed p-values’ and the Y-axis shows the ‘expected p-values’. (TIFF)

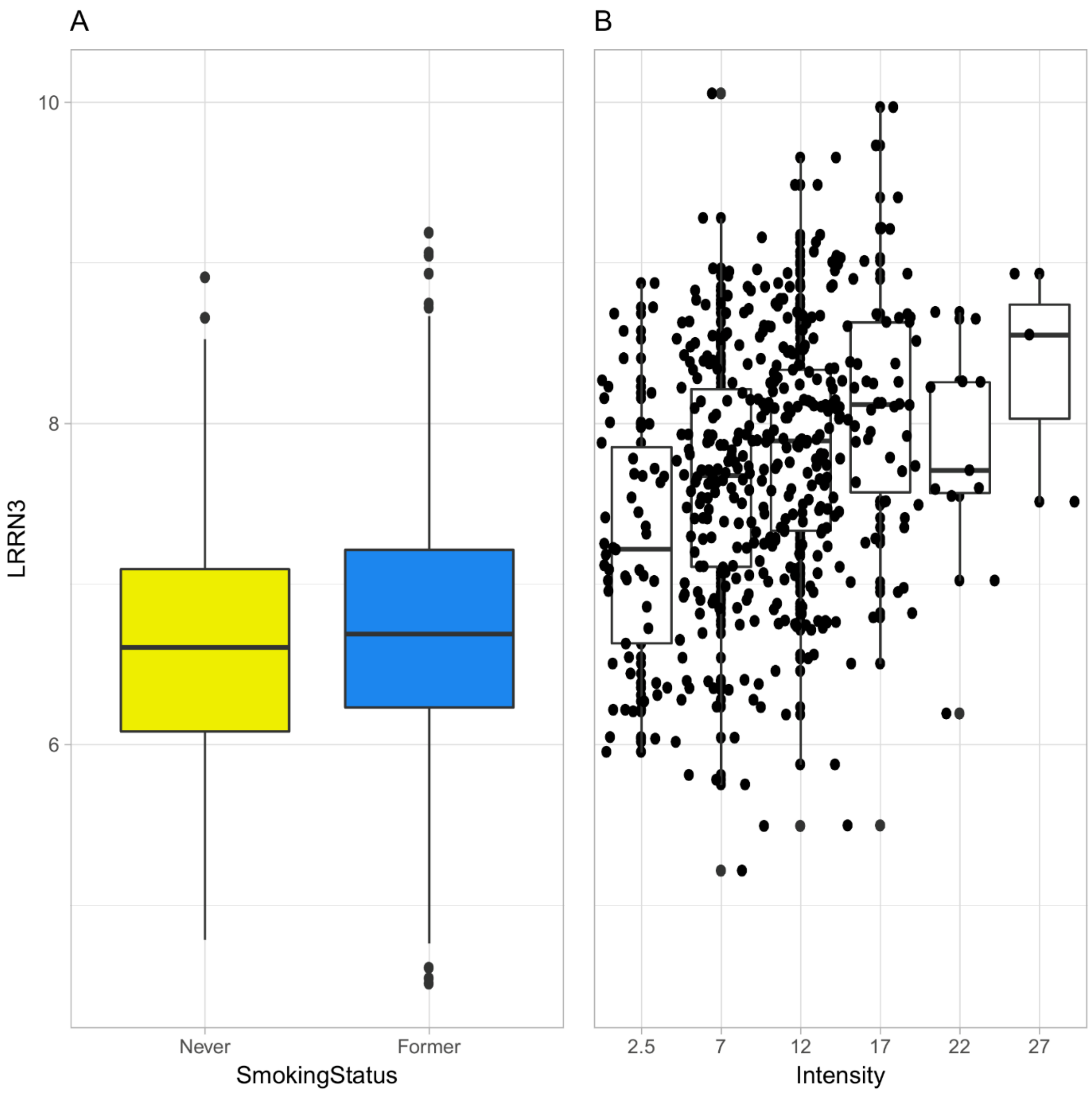

Supplementary Figure S5. Distributions of expression values for the top-ranked significant gene (*LRRN3*) (A) among never and former smokers and (B) among current smokers according to smoking intensity. In figure A: yellow colour represents never smokers and blue colour represents former smokers; boxes extend from the 25th to the 75th percentile, horizontal bars represent the median, whiskers extend 1.5 times the length of the interquartile range above and below the 75th and 25th percentiles, respectively, and outliers are represented as points. (TIFF)

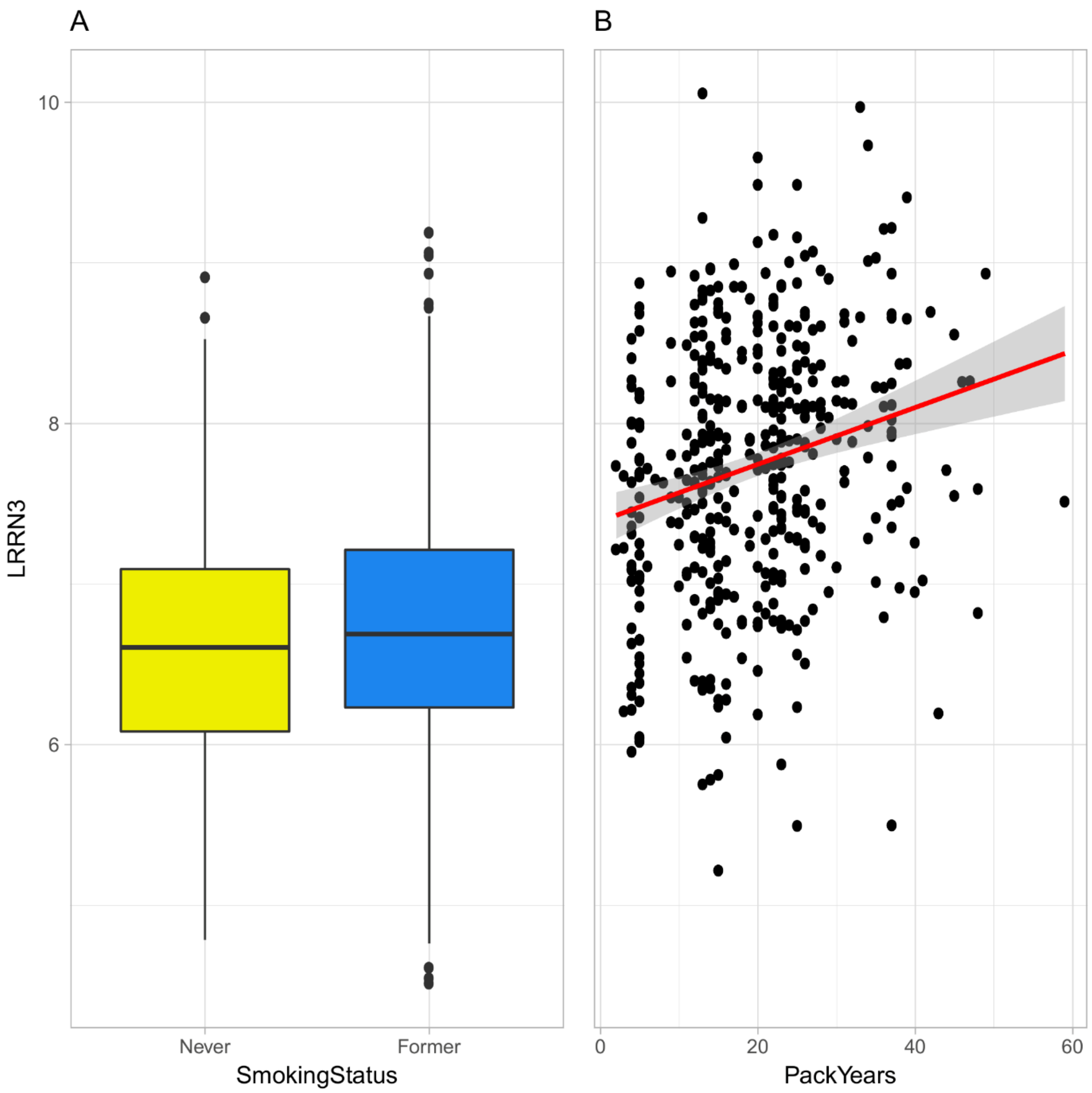

Supplementary Figure S6. Distributions of expression values for the top-ranked significant gene (*LRRN3*) (A) among never and former smokers and (B) among current smokers according to pack-years. In figure A: yellow colour represents never smokers and blue colour represents former smokers; boxes extend from the 25th to the 75th percentile, horizontal bars represent the median, whiskers extend 1.5 times the length of the interquartile range above and below the 75th and 25th percentiles, respectively, and outliers are represented as points. In figure B: the red line represents the regression line with a shaded grey area representing the standard error. (TIFF)

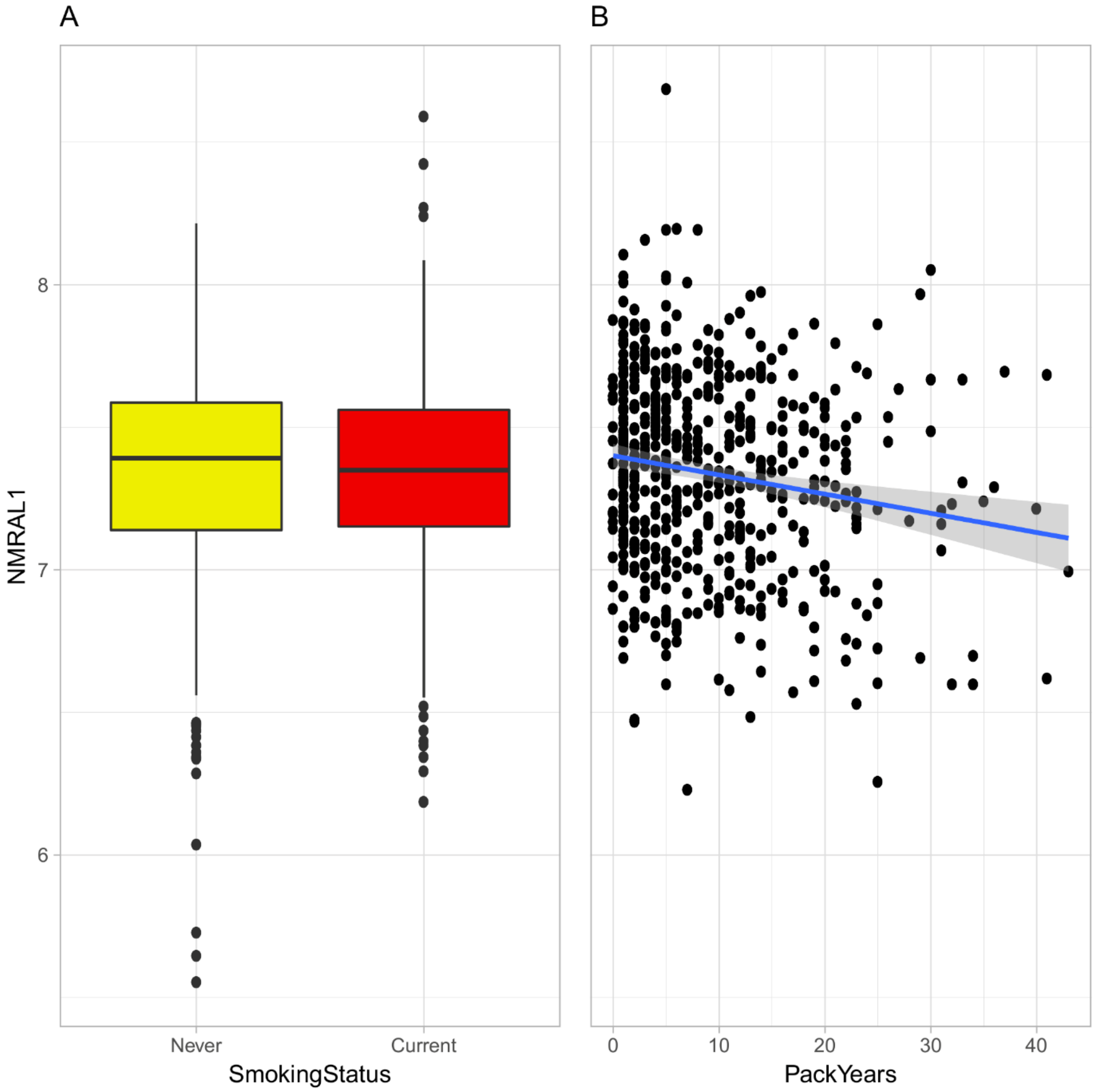

Supplementary Figure S7. Distributions of expression values for the top-ranked significant gene (*NMRAL1*) (A) among never and current smokers and (B) among former smokers according to pack-years. In figure A: yellow colour represents never smokers and red colour represents current smokers; boxes extend from the 25th to the 75th percentile, horizontal bars represent the median, whiskers extend 1.5 times the length of the interquartile range above and below the 75th and 25th percentiles, respectively, and outliers are represented as points. In figure B: the blue line represents the regression line with a shaded grey area representing the standard error. (TIFF)

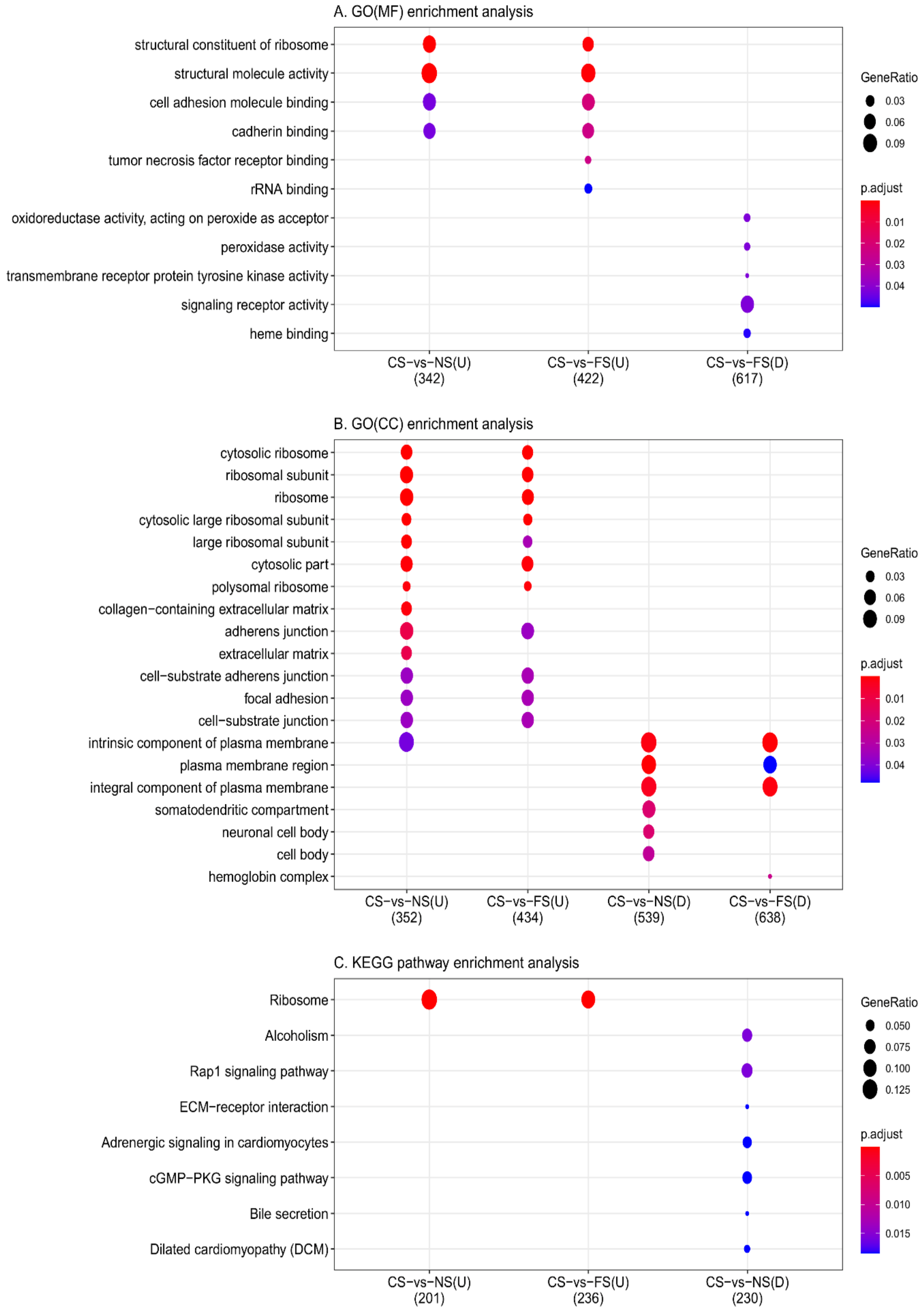
A. GO(MF) enrichment analysis
structural constituent of ribosome
structural molecule activity
cell adhesion molecule binding
cadherin binding
tumor necrosis factor receptor binding
rRNA binding
oxidoreductase activity, acting on peroxide as acceptor
peroxidase activity
transmembrane receptor protein tyrosine kinase activity
signaling receptor activity
heme binding
CS−vs−NS(U) (342)
CS−vs−FS(U) (422)
CS−vs−FS(D) (617)
GeneRatio
0.03
0.06
0.09
p.adjust
0.01
0.02
0.03
0.04
B. GO(CC) enrichment analysis
cytosolic ribosome
ribosomal subunit
ribosome
cytosolic large ribosomal subunit
large ribosomal subunit
cytosolic part
polysomal ribosome
collagen−containing extracellular matrix
adherens junction
extracellular matrix
cell−substrate adherens junction
focal adhesion
cell−substrate junction
intrinsic component of plasma membrane
plasma membrane region
integral component of plasma membrane
somatodendritic compartment
neuronal cell body
cell body
hemoglobin complex
CS−vs−NS(U) (352)
CS−vs−FS(U) (434)
CS−vs−NS(D) (539)
CS−vs−FS(D) (638)
GeneRatio
0.03
0.06
0.09
p.adjust
0.01
0.02
0.03
0.04
C. KEGG pathway enrichment analysis
Ribosome
Alcoholism
Rap1 signaling pathway
ECM−receptor interaction
Adrenergic signaling in cardiomyocytes
cGMP−PKG signaling pathway
Bile secretion
Dilated cardiomyopathy (DCM)
CS−vs−NS(U) (201)
CS−vs−FS(U) (236)
CS−vs−NS(D) (230)
GeneRatio
0.050
0.075
0.100
0.125
p.adjust
0.005
0.010
0.015

Supplementary Figure S8. Summary of functional enrichment analyses for up- and down-regulated genes for the (A) GO(MF), (B) GO(CC), and (C) KEGG pathway databases. The colour of the dots indicates the adjusted p-value, where red dots represent the most enriched categories; the 'GeneRatio' indicates the proportion of genes overlapping between lists of differentially expressed genes (DEGs) and the genes in gene ontology categories. GO: gene ontology; MF: molecular functions; CC: cellular components; KEGG: Kyoto Encyclopedia of Genes and Genomes; CS-vs-NS: comparison of current smokers vs never smokers; CS-vs-FS: comparison of current smokers vs former smokers; U: Up-regulated genes; D: Down-regulated genes.
